# Identification of lncRNA functions in lung cancer based on associated protein-protein interaction modules

**DOI:** 10.1038/srep35939

**Published:** 2016-10-27

**Authors:** Chih-Hsun Wu, Chia-Lang Hsu, Pei-Chun Lu, Wen-Chang Lin, Hsueh-Fen Juan, Hsuan-Cheng Huang

**Affiliations:** 1 Institute of Biotechnology in Medicine, National Yang-Ming University, Taipei 112, Taiwan; 2 Institute of Biomedical Informatics, Center for Systems and Synthetic Biology, National Yang-Ming University, Taipei 112, Taiwan; 3 Department of Life Science, National Taiwan University, Taipei 106, Taiwan; 4 Institute of Biomedical Sciences, Academia Sinica, Taipei 115, Taiwan; 5 Graduate Institute of Biomedical Electronics and Bioinformatics, Institute of Molecular and Cellular Biology, National Taiwan University, Taipei 106, Taiwan

## Abstract

Long non-coding RNAs (lncRNAs) have been found to play important roles in various biological processes; however, many of their functions remain unclear. In this study, we present a novel approach to identify the lncRNA-associated protein-protein interaction (PPI) modules and ascertain their functions in human lung squamous cell carcinoma. We collected lncRNA and mRNA expression profiles of lung squamous cell carcinoma from The Cancer Genome Atlas. To identify the lncRNA-associated PPI modules, lncRNA-mRNA co-expression networks were first constructed based on the mutual ranks of expression correlations. Next, we examined whether the co-expressed mRNAs of a specific lncRNA were closely connected by PPIs. For this, a significantly connected mRNA set was considered to be the lncRNA-associated PPI module. Finally, the prospective functions of a lncRNA was inferred using Gene Ontology enrichment analysis on the associated module. We found that lncRNA-associated PPI modules were subtype-dependent and each subtype had unique molecular mechanisms. In addition, antisense lncRNAs and sense genes tended to be functionally associated. Our results might provide new directions for understanding lncRNA regulations in lung cancer. The analysis pipeline was implemented in a web tool, available at http://lncin.ym.edu.tw/.

Long non-coding RNAs (lncRNAs), more than 200 nucleotides in length but without protein-coding capacity, are a novel class of mRNA-like transcripts. They function in diverse cellular contexts by regulating chromatin structure and gene expression. lncRNAs function as epigenetic and transcriptional regulators by acting as scaffolds for the assembly of chromatin- and gene-regulating complexes, or as guides directing other regulators to specific sites in the genome, resulting in the activation or repression of gene expression^1^,^2^,^3^,^4^. Furthermore, lncRNAs can alter the post-transcriptional regulation of mRNA, cellular signaling, and protein activity through allosteric regulation^4^,^5^. Some lncRNAs can act as microRNA sponges to reduce the amount of microRNA available to target genes^6^,^7^,^8^. To obtain a better understanding of regulatory networks in cell, it is important to understand the function of each lncRNA.

According to the latest version of GENCODE annotation (v24), more than 15,000 lncRNAs have been identified; however, the biological and molecular characteristics of the large majority remain unknown. To accelerate the study of lncRNA, many computational methods have been proposed for functional predictions. Current methods for the annotation of lncRNA functions rely on their association with protein-coding genes using gene co-expression^9^,^10^ and the competing endogenous RNA hypothesis^11^. However, these methods might associate lncRNA with many coding genes, which are not functionally related and this could result in high rate of false positives.

To address this issue, we have developed a new computational pipeline to annotate lncRNA functions based on associated mRNA co-expression and protein-protein interaction (PPI) networks. Proteins do not function independently, but interact with others to mediate signaling pathways, cellular processes, and organismal systems; hence, PPI information can aggregate functionally related genes to a functional module. In our previous studies, an integration of gene expression and protein-protein interactions was successfully utilized to uncover the functions of microRNAs in various cancers^12^,^13^. Therefore, PPI information might be also useful for investigating the function of lncRNAs.

To demonstrate the capacity of our proposed method, we investigated lncRNAs in lung cancer. Lung cancer is the main cause of cancer-related deaths worldwide. Based on histology, lung cancers can be classified as either small cell lung cancer (SCLC) or non-small cell lung cancer (NSCLC). NSCLC makes up to 80% of lung cancers and accounts for the majority of cancer deaths worldwide; therefore, we focused on NSCLC. Recent studies have indicated that the abnormal expression of lncRNAs influences tumorigenesis and plays both oncogenic and tumor suppressive roles^14^,^15^,^16^ and suggested that lncRNAs could serve as diagnostic biomarkers and therapeutic targets in lung cancer^17^. In this study, we analyzed the mRNA expression datasets of the NSCLC human lung squamous cell carcinoma (LUSC) obtained from The Cancer Genome Atlas (TCGA) project, and found that several lncRNAs may play critical roles in the tumorigenesis of different LUSC subtypes.

## Results and Discussion

### Overview of the analysis pipeline

**Figure 1:**
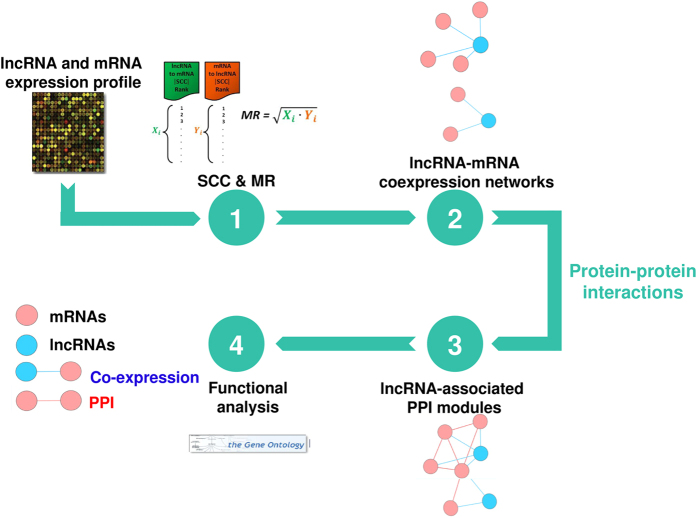
The flowchart of lncRNA-associated PPI module identification For a given lncRNA and mRNA expression profiles, the correlations between each pair (lncRNA and mRNA) were measured using the Spearman’s correlation coefficient (SCC). The co-expressed mRNAs were then determined using the mutual correlation rank score (MR), the geometric average of the absolute value of SCC (|SCC|) rank from lncRNA to mRNA and vice versa. For each lncRNA, the top-scoring mRNAs were selected as the co-expressed mRNAs (lncRNA-associated mRNAs). Subsequently we used protein-protein interaction analysis of co-expression mRNAs to find lncRNA-associated PPI modules. Finally, we performed gene ontology enrichment analysis on the associated mRNAs of each lncRNA module to understand their regulatory functions.

To increase the understanding of lncRNA functions, we proposed a novel computational method to predict functions by identifying lncRNA-associated modules in protein-protein interaction networks. The analysis pipeline is depicted in Fig. 1. Firstly, lncRNA-mRNA co-expression networks were constructed. The co-expression level between lncRNA and mRNA was calculated using the Spearman correlation coefficient (SCC), as it is less sensitive to outliers. The mutual rank (MR) index was used to define the co-expressed lncRNA-mRNA pairs because a pair with low expression similarities might work together if no other mRNAs are highly co-expressed. It has been documented that MR is a better measure of similarity than the correlation value in order to identify related genes^18^. The top-scoring mRNAs were selected as the co-expressed mRNAs for each lncRNA. Subsequently we examined whether there were a significant number of pairs among lncRNA-co-expressed mRNAs connected by PPIs using a permutation test. The significantly connected subset of lncRNA-co-expressed mRNAs was defined as the lncRNA-associated PPI module. Finally, we performed gene ontology (GO) enrichment analysis on the lncRNA modules to understand the regulatory functions.

### Construction of the lncRNA-mRNA co-expression network in lung squamous cell carcinoma

We obtained 113 LUSC tumor samples from TCGA project. According to the re-annotation of the Affymetrix exon array probes^19^, this dataset consisted of 10,207 lncRNAs and 18,319 mRNAs. The top 0.1% of lncRNA-mRNA pairs based on MR index (i.e. 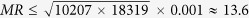) were considered as co-expressed pairs. A total of 99,175 co-expressed lncRNA-mRNA pairs consisting of 10,063 lncRNAs and 18,207 mRNAs were determined. On average, each lncRNA had 9.8 co-expressed mRNAs (Figure S1).

### lncRNA-co-expressed mRNAs tended to be connected by PPI

**Figure 2:**
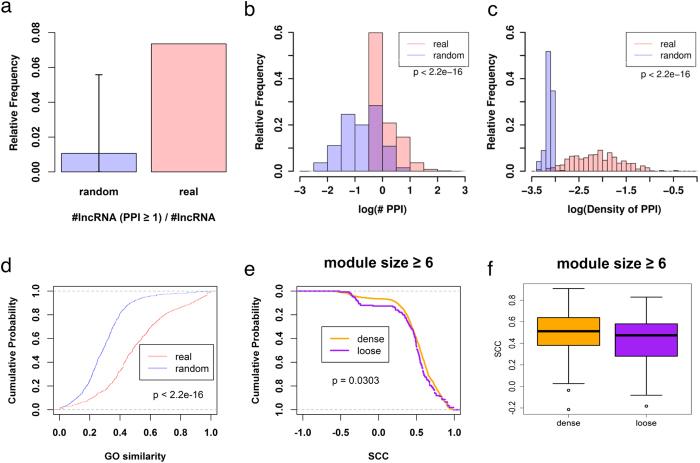
Functional and correlation analysis of lncRNA-associated PPI modules. (**a**) Frequency of lncRNAs which co-expressed mRNAs are connected by at least one PPI in real (red) and random (blue) co-expression networks. (**b**) Distributions of mean number of PPIs for co-expressed mRNAs of each lncRNA in real (red) and random (blue) co-expression networks. Purple area is the overlap of the two distributions. (**c**) Distributions of the mean density of PPIs in real (red) and random (blue) co-expression networks. Purple area is the overlap of the two distributions. (**d**) Cumulative density plots for GO similarity between mRNA-mRNA pairs which are both co-expressed with a given lncRNA and connected via PPIs (red) and random pairs (blue). The p-value was calculated by the KS test. (**e**) Cumulative density plots for two SCC distributions of 1,257 mRNA-mRNA pairs in dense modules and 151 mRNA-mRNA pairs in loose modules. The *p*-value was calculated by KS test. (**f**) The mean of SCC for all mRNA-mRNA pairs in 106 dense modules and 33 loose PPI modules.

Among the 10,063 lncRNAs, the co-expressed mRNAs of only 740 lncRNAs were connected by at least one PPI. To examine whether the number of PPIs connecting co-expressed mRNAs of a given lncRNA was observed by chance, the real lncRNA-mRNA co-expression network was compared to 1,000 random co-expression networks whose topological properties were identical to the real one, but mRNAs were re-sampled. We found that the frequency of lncRNAs with co-expressed mRNAs connected by at least one PPI in real co-expression networks (7%) was higher than that in random co-expression networks (Fig. 2a). Then we compared the distribution of mean number of PPIs of 740 lncRNAs in real and random co-expression networks, and found the mRNAs co-expressed with a lncRNA have significantly higher number of connections via PPIs (*p* < 2.2 × 10^−16^, Fig. 2b). Because the number of co-expressed mRNAs may affect the observed number of PPIs, we also examined the distribution of interaction density of 740 lncRNAs and found that the interaction density of these lncRNAs was still significantly higher than that in random co-expression networks (*p* < 2.2 × 10^−16^, Fig. 2c). These results indicated that the co-expressed mRNAs for a lncRNA tended to be connected by PPIs.

Next, we examined whether the mRNAs which are co-expressed with a lncRNA and connected by PPIs tend to be involved in similar biological processes. The co-expressed mRNAs without any PPI connections were ignored. There were 1,653 mRNAs connected by 2,264 PPIs. The functional similarity scores of these 2,264 pairs were calculated using the R package GoSemSim^20^. Random pairs were generated by permutating these 2,264 PPI pairs and the functional similarity scores of these random pairs were calculated as a background distribution. In comparison with the background distribution, the functional similarities among PPIs from lncRNA-co-expressed mRNAs were significantly high (*p* < 2.2 × 10^−16^, Fig. 2d). These indicated that integrating co-expression and PPI information might be useful for identifying the associated functions of a lncRNA.

### Identification of lncRNA-associated PPI modules in generic and subtypes of lung squamous cell carcinoma

The 740 lncRNAs whose co-expressed mRNAs were connected by at least one PPI were further analyzed to identify the lncRNA-associated PPI modules. A permutation test was used to assess whether the co-expressed mRNAs of a lncRNA interacted with each other more frequently than expectation. If the number of PPIs among the lncRNA-co-expressed mRNAs were significantly higher (*p* < 0.05), they are defined as a dense lncRNA-associated PPI module. Otherwise, they are called a loose module. There were 466 lncRNAs whose co-expressed mRNAs form dense modules in the PPI network. To demonstrate that these lncRNA-associated dense modules were biologically meaningful, the expression correlations and functional similarities among components of the modules were assessed. We hypothesized that the dense modules would tend to have higher expression correlations and functional similarities than loose modules. In addition, the module sizes were also considered because the modules with fewer components may not have had sufficient biological functions for further analysis. With different module size thresholds, we compared the distribution of the expression correlation and functional similarities between the identified dense and loose modules (Figures S2 and S3). Using a threshold of module size ≥ 6, 1,257 mRNA-mRNA pairs in dense modules had a significantly higher expression correlation than 151 mRNA-mRNA pairs in loose modules (*p* = 0.03, Fig. 2e). The SCC mean of all the mRNA-mRNA pairs in the 106 dense modules was also higher than that of the 33 loose modules (Fig. 2f). Moreover, the mRNAs in dense modules with six or more co-expressed mRNAs had significantly higher functional similarities than those in loose modules with an identical component size threshold (*p* = 0.0017, Figure S3). To brevity we shall refer to the dense module as the lncRNA-associated PPI module. Therefore, using a threshold module size of six, we finally obtained 106 lncRNA-associated PPI modules.

We were also interested in the subtype-specific lncRNA-associated PPI modulate in LUSC. There are four main gene expression subtypes for LUSC: the classical, basal, secretory, and primitive subtypes. Each subtype has different molecular and clinical characteristics^21^,^22^. To identify the subtype-specific lncRNA-associated PPI modules, we applied the same analysis approach on samples from each subtype. In these datasets, there were 31 classical, 25 basal, 20 secretory, and 13 primitive subtype samples, while the remaining 24 samples were unknown. The primitive lung cancer samples were excluded for further analysis due to their small sample size. Using the same criteria (permutation test *p* < 0.05 and module size ≥ 6), there were 48, 50, and 53 lncRNA-associated PPI modules identified in classical, basal, and secretory LUSC, respectively.

### The associated modules revealed the functions of well-studied lncRNAs

The functional enrichment analysis was performed on each lncRNA-associated PPI module. The enriched functions (corrected *p* < 0.05) for each lncRNA-associated PPI module in generic and each subtype LUSC can be found in the Lncin website (http://lncin.ym.edu.tw/Case_Lung/Lung_result.php?id=cancer_lung#Predict).

**Figure 3:**
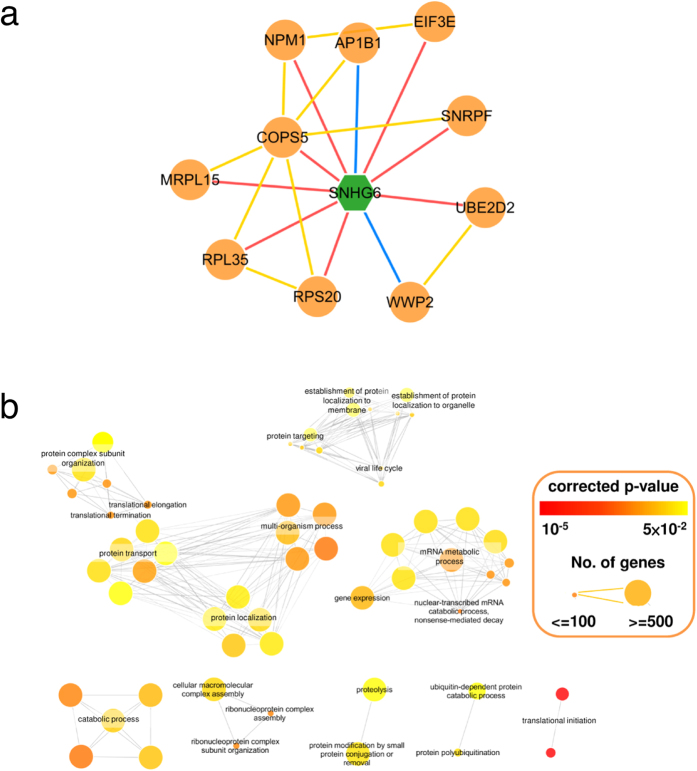
SNHG6-associated PPI module and its enriched functions in generic LSCC. (**a**) The SNHG6-associated PPI module is visualized as a graph. Hexagon and oval node denote lncRNA and mRNA. Edge color represents different interaction type. (**b**) Enriched GO terms derived from the SNHG6-associated PPI module are visualized as a network. Nodes represent enriched GO terms (corrected *p* < 0.05) and links between the nodes represent the overlap score calculated from the number of genes two GO terms share (threshold = 0.85). Node color encodes the statistical significance of enrichment analysis. The node size is proportional to the number of genes belonging to the corresponding GO term.

To demonstrate the capability of our analysis pipeline, we investigated 18 well-studied lncRNAs and found that our method could reveal the involved functions of these lncRNAs in most cases (Table S1). For example, SNHG6 is associated with ribosomes and is relatively resistant to nonsense-mediated mRNA decay^23^. The SNHG6-associated PPI module in generic LUSC was illustrated in Fig. 3a. The genes in the generic SNHG6-associated PPI module were enriched in ribosome-related functions (corrected *p* < 0.05, Fig. 3b). MEG3 is important for proper growth and development, and is considered to be a putative tumor suppressor through the activation of p53 and inhibition of cell proliferation^24^,^25^. The functional analysis of MEG3-associated modules identified in classical LUSC revealed that the majority of MEG3-associated genes were related to extracellular structure organization, cell migration, cell adhesion, and growth (Figure S4), consistent with previous studies. MALAT1 has been considered to be a oncogene that promotes tumorigenesis^26^ and regulates gene expression by altering the histone modifications on chromatin^27^. In our analysis, the MALAT1-associated module was identified in classical LUSC, and the components of this module were enriched in chromatin modification functions (Figure S5). TUG1 has been reported to promote cell proliferation in many cancer types, including osteosarcoma^28^, non-small cell lung cancer^29^, and esophageal squamous cell carcinoma^30^. TUG1 also promotes migration in esophageal squamous cell carcinoma^30^. In our study, the TUG1-associated module was identified in classical LUSC, and the function analysis showed that TUG1 functions were related to the regulation of cell proliferation, positive regulation of growth, regulation of the cell cycle, and regulation of cell adhesion (Figure S6).

Although our method could reveal the prospective functions of lncRNA functions mostly, it failed in a few cases. For example, FAM3D-AS1 is involved in the migration and cell proliferation in head and neck squamous cell carcinoma^31^, but our inferred functions in basal LUSC are RNA splicing and peptidyl-amino acid modification (Table S1). The inconsistency might be due to the difference in cancer cell types since lncRNAs may play different roles in different cell lines, tissues and cancers. In addition, the incomplete GO and PPI information could influence the effectiveness of our approach.

### Functions of lncRNA-associated PPI modules in generic lung squamous cell carcinoma

**Figure 4:**
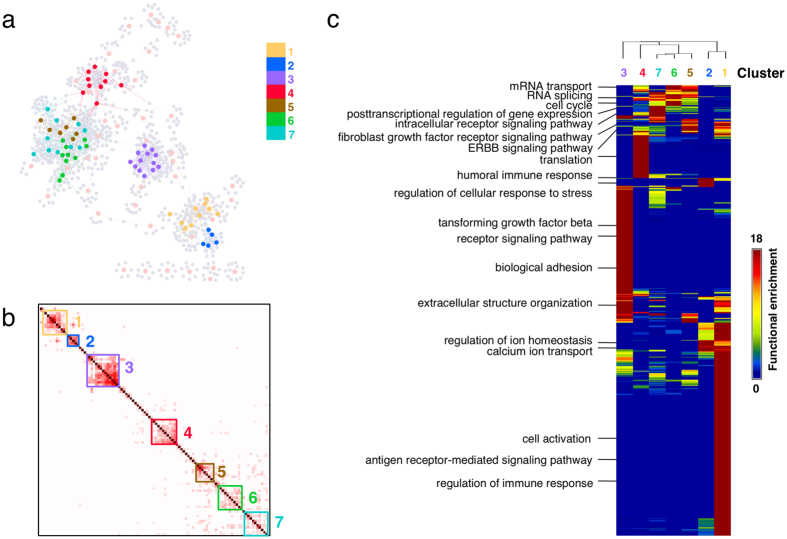
Generic lncRNA-associated PPI modules, the similarity of lncRNAs, and the functions of seven clusters in lung cancer. (**a**) The assembled networks of all lncRNA-associated modules were visualized as graph. The gray nodes represent the mRNAs, the remaining nodes represent the lncRNAs. The different colors correspond to different network clusters from (**b**). The gray lines represent the PPIs among these lncRNA-co-expressed mRNAs, and the remaining lines represent the co-expressed pairs of lncRNAs and mRNAs. (**b**) Clustering of the lncRNAs in the largest subnetwork in (**a**). The similarity of lncRNAs was determined using the common lncRNA-co-expressed mRNAs. The hierarchical clustering with average linkage was performed using GAP software. The lncRNAs were classified into seven clusters. (**c**) Comparison of enriched biological functions among seven lncRNA clusters. The GO terms which are significantly enriched (corrected *p* < 0.05) in at least one of clusters were retained, and the *p*-values of each GO term in all clusters were converted to −log_10_ *p* as the raw matrix for visualization. The heatmap and dendrogram plot showing similarity between lncRNA clusters were generated using GAP software with Pearson correlation and complete linkage.

The assembled network of 106 lncRNA-associated PPI modules in generic LUSC revealed that the majority of lncRNAs were connected through common co-expressed mRNAs or PPIs (Fig. 4a). We performed GO enrichment analysis of all the lncRNA-associated mRNAs to investigate the possible biological functions of lncRNAs in overall generic lung cancer. The enriched functions (corrected *p* < 0.05) included multi-organism processes, macromolecular complex subunit organization, mRNA metabolic processes, gene expression, extracellular structure organization, cellular component disassembly, and immune system processes.

Although the majority of lncRNAs were in the largest connected component, this network could be broken down to subnetworks. The distance matrix of lncRNAs was generated by calculating the overlap of lncRNA-co-expressed mRNAs. Hierarchical clustering was performed to break down the largest connected component of the network. After the removal of clusters with less than five lncRNAs, seven clusters were identified (Fig. 4b). The GO enrichment analysis on these seven clusters revealed that lncRNAs in each cluster might be associated with specific functions (Fig. 4c). The lncRNA modules in Cluster 1 were enriched with biological processes such as cell activation, the antigen receptor-mediated signaling pathway, and regulation of the immune response. Cluster 2 was enriched with the humoral immune response, regulation of ion homeostasis, calcium ion transport, and regulation of the cellular response to stress, including positive regulation of the stress-activated MAPK cascade and positive regulation of the JNK cascade. Cluster 3 was enriched with extracellular structure organization and biological adhesion, and Cluster 4 was enriched with translation. Cluster 5 was enriched with the fibroblast growth factor receptor-signaling pathway and the ERBB signaling pathway. Cluster 6 was enriched with mRNA transport, RNA splicing, and the cell cycle. Cluster 7 was enriched with the post-transcriptional regulation of gene expression and intracellular receptor signaling pathways. The results indicate that lncRNAs might play diverse roles in generic LUSC samples.

### The comparison and functions of subtype-specific lncRNA-associated PPI modules

**Figure 5:**
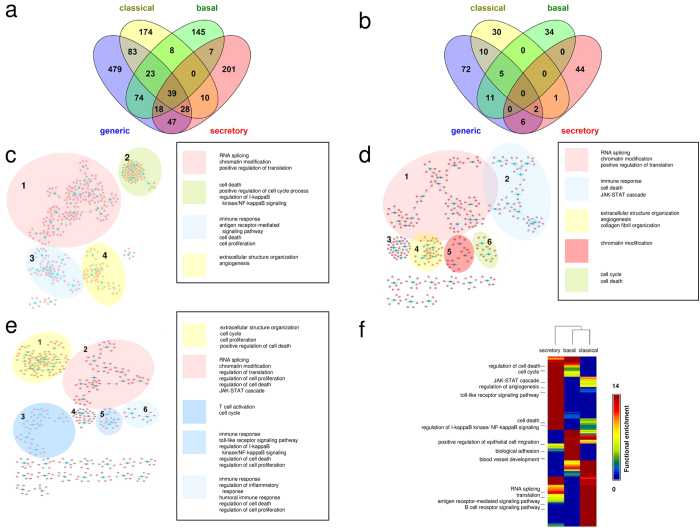
lncRNA-associated PPI modules and their functions in different lung cancer subtypes. (**a**) Venn diagram showing the number of mRNAs in lncRNAs-associated modules derived from the overall generic lung cancer dataset, as well as the lung cancer subtypes, including classical, basal, and secretory. (**b**) Venn diagram showing the number of lncRNAs in lncRNAs-associated modules derived from generic lung cancer and each lung cancer subtype. (**c–e**) The assembled network of lncRNA-associated PPI modules identified in the classical (**c**), basal (**d**), and secretory (**e**) subtype of lung cancers. The blue and red nodes denote lncRNAs and mRNAs, respectively. The lncRNAs which are connected via co-expressed mRNAs or PPI were highlighted in colors and their enriched functions (Fisher’s exact test, corrected *p* < 0.05) are described in the box. (**f**) Comparison of enriched biological functions among three subtypes of lung cancer. The GO terms which are significantly enriched (corrected *p* < 0.05) in at least one of subtypes were retained, and the *p*-values of each GO term in all clusters were converted to −log_10_ *p* as the raw matrix. The heatmap and dendrogram plot showing similarity between subtypes were generated using GAP software with Pearson correlation and complete linkage.

The comparison of lncRNAs-associated modules derived from each subtype and generic samples revealed that the overlap of co-expressed mRNAs and lncRNAs among different subtypes were quite low (Fisher’s exact test, left-sided *p* < 0.02, Fig. 5a,b). In addition, there were 35 lncRNAs which associated PPI modules were identified in at least two subtypes, but the components of the subtype-specific module of a given lncRNA were different (Table S2). Interestingly, we examined the functional similarity between modules associated by a given lncRNA in different subtypes, and 13 of lncRNAs display significantly high in functional similarity scores (functional similarity > 0.8, Table S2). This suggests that some lncRNAs may play similar roles in different subtype LUSC by regulating different genes, while some of them play quite different roles in each subtype.

The integrative network of the identified modules in each subtype of LUSC showed that some lncRNA-associated PPI modules could connect to form a large module (Fig. 5c–e). We performed the GO enrichment analysis on clusters, which contained at least two lncRNA-associated PPI modules, to determine lncRNA-involved biological processes. We found that some functions, such as RNA splicing and chromatin modification, were enriched in all three subtypes (corrected *p* < 0.05) and that each LUSC subtype had unique molecular mechanisms. For example, most lncRNAs identified in secretory LUSC might be involved in immune-related signaling pathways, including the JAK-STAT cascade, regulation of I-kappaB kinase/NF-kappaB signaling, and the toll-like receptor signaling pathway (Fig. 5f), consistent with a previous study that suggested secretory LUSC is related to immune system processes^21^. In addition, the secretory LUSC-specific lncRNAs were uniquely related to the processes of cell proliferation, cell death, and cell cycle function. Previous work has indicated that secretory-type cell lines were less sensitive to the most effective anticancer drugs, due to low proliferation activity^32^. Our investigation suggests that these lncRNAs might be associated with the low drug-sensitivity of secretory LUSC.

### Antisense lncRNAs tend to play similar roles as sense genes

**Figure 6:**
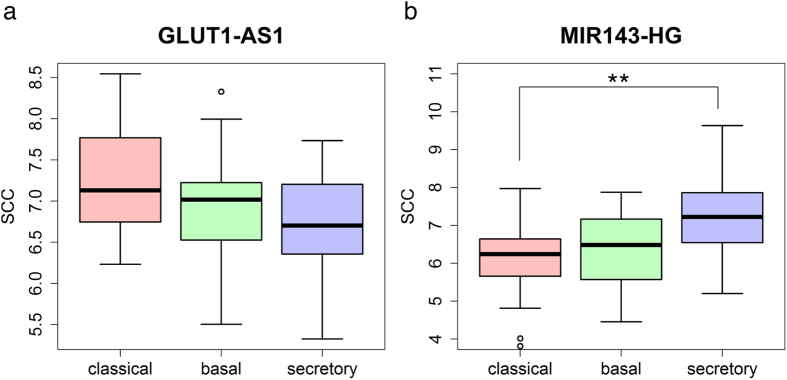
Comparison of the expression of GLUT1-AS1 and MIR143HG among lung cancer subtypes. (**a**) The expression levels of GLUT1-AS1 in classical (red), basal (green), and secretory (blue) lung cancer subtypes. (**b**) The expression levels of the MIR143 host gene in classical (red), basal (green), and secretory (blue) lung cancer subtypes. The *p*-value was calculated by the KS test (**p < 0.01).

Many identified lncRNAs are located on the antisense strand of DNA opposing a known gene in the genome. Because some antisense lncRNAs modulate the expression of sense genes^33^, we were interested in whether antisense lncRNAs participate in the same functions as the sense genes. To address this, we examined several antisense lncRNAs. GLUT1-AS1 is the tail-to-tail antisense of the glucose transporter 1 (GLUT1) which is selectively essential for CD4 T cell activation^34^. The enriched functions of the GLUT1-AS1-associated module in secretory LUSC included positive regulation of T cell activation and immune effector processes (Figure S7). We also found that GLUT1-AS1 was negatively correlated with their associated genes (Figure S7), and had lower expression in secretory LUSC compared with other subtypes (Fig. 6a). TBA5-AS1 is the tail-to-tail antisense of T-box 5 (TBX5) which is a transcription factor and is associated with lung agenesis^35^. Our results showed that the components of the TBX5-AS1-associated module in classical LUSC were significantly enriched in extracellular structure organization (Figure S8). BHLHE40-AS1 is the tail-to-tail antisense of the transcription factor basic helix-loop-helix family member e40 (BHLHE40). BHLHE40 is negatively related with the cancer TNM stage classification and inhibits proliferation in NSCLC^36^. Our results showed that the function of the BHLHE40-AS1-associated module in secretory LUSC was related to the regulation of cell proliferation and cell adhesion (Figure S9). These examples reveal that antisense lncRNAs and sense genes tend to be involved in similar biological processes.

### miR-143 and its host lncRNA tend to play similar roles

In our analysis, we identified some host lncRNAs of microRNAs–associated module, such as MIR143 host gene (MIR143HG)–associated module was recognized in the secretory LUSC subtype and miR-143 located at the exon of the MIR143HG, so we assessed whether there was any functional association between a microRNA and its host lncRNA. The MIR143HG–associated module was only identified in the secretory LUSC subtype, and indeed, the expression of MIR143HG in the secretory subtype was significantly higher compared with the classical subtype (*p* = 0.0056, Fig. 6b). The expression of mRNAs in the MIR143HG-associated PPI module in secretory LUSC were all positively correlated with MIR143HG, and these mRNAs were enriched in functions related to extracellular matrix organization and cell adhesion (Figure S10). Many studies have reported that the overexpression of miR-143 inhibited cell migration and invasion^37^,^38^. Hence, MIR143HG and miR-143 might be involved in similar biological processes via different regulatory mechanisms, whereby MIR143HG activates targets and miR-143 represses targets.

## Conclusions

Because the biological and molecular characteristics of the most lncRNAs remain unknown, this work presents a new computational pipeline for functional annotation of lncRNAs. Our method utilized not only the lncRNA-mRNA co-expression networks based on the rank of correlation which is a better measure of similarity than the correlation value, but also protein-protein interactions among co-expressed mRNAs to identify a set of mRNAs that may be modulated by lncRNA. Our analysis results revealed that the co-expressed mRNAs for a lncRNA tended to be connected by PPIs and the functions of a lncRNA could be inferred from its connected co-expressed mRNAs. We implemented a practical tool, named “Lncin”, for our methodology, and it is freely available at http://lncin.ym.edu.tw/. It provides a user-friendly interface for non-bioinformatics experts to determine the lncRNA-associated PPI modules and investigate the unknown or novel functions of lncRNAs.

For well-studied lncRNAs, our method could express their prospective functions to a certain extent. For antisense lncRNAs, our analysis indicates that these lncRNAs and their antisense genes tend to be involved in similar biological processes. Similarly, miR-143 and its host lncRNA tend to play similar roles. These findings might provide new directions to further understand the lncRNA regulation based on the associated PPI modules.

We applied this method to LUSC dataset and found that lncRNA-associated PPI modules are subtype-dependent because the overlap of co-expressed mRNAs and lncRNAs among different subtypes were quite low. In addition, the predicted functions of lncRNAs revealed that each LUSC subtype might be associated with different pathogenesis mechanisms. We also identified several lncRNAs might play critical roles in the tumorigenesis of different LUSC subtypes. Although a few common lncRNAs were identified in different cancer subtypes, the components of associated PPI module were quite different between subtypes. The common lncRNAs may play different roles in different subtypes by regulating different genes, but some of them play similar functional roles in different subtypes. These subtype-specific lncRNAs might be useful for understanding subtype-specific carcinogenesis and developing subtype-specific treatment strategies.

## Materials and Methods

### lncRNA and mRNA expression profiles

The lncRNA and mRNA expression profiles for LUSC were collected from TCGA^19^. According to the re-annotation of microarray probes^19^, this expression dataset consisted of 10,207 lncRNAs and 18,319 mRNAs.

### Construction of the lncRNA-mRNA co-expression networks

The lncRNA-mRNA co-expression networks were constructed based on the rank of correlations. The correlations between lncRNAs and mRNAs were evaluated by the Spearman correlation coefficients (SCC) and their absolute values |SCC| were used to rank lncRNAs and mRNAs. For each pair, mRNA A and lncRNA B, the mutual rank (MR) index was calculated as a geometrically average of the SCC rank from A to B and that from B to A as follows:


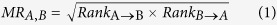


To reduce the false discovery rate, we considered the top 0.1% of pairs to be co-expressed, namely 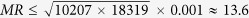.

### Identification of lncRNA-associated PPI modules

The protein-protein interaction data were collected from the following databases: MINT^39^, BioGRID^40^, DIP^41^, HPRD^42^, and IntAct^43^. The protein identifiers of each database were mapped using the Entrez Gene ID^44^. There were 15,472 proteins and 143,300 interactions in total after removing self- and duplicate interactions.

The selected lncRNAs whose co-expressed mRNAs were connected by at least one PPI were further examined using the following permutation test. For a lncRNA with *n* co-expressed mRNAs connected by *x* PPIs, we randomly selected a set of *n* mRNAs from the pool of 18,319 mRNAs and counted the number of PPIs connected among this set of randomly selected mRNAs. We repeated this procedure for 1,000 times and calculated the mean (μ) and standard deviation (σ) of the numbers of PPIs in the 1,000 random mRNA sets. The observed value *x* was converted to z-score by 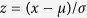, and the corresponding *p*-value can be obtained. If *p* < 0.05, the lncRNA and its co-expressed mRNAs connected by PPIs were defined as a dense lncRNA-associated PPI module. Otherwise, if *p* > 0.05, they were called a loose module.

We further disregarded the modules with less than six co-expressed mRNAs since they could fail in functional enrichment analysis. Finally, 106, 48, 50, and 53 lncRNA-associated PPI modules were recognized in generic, classical, basal, and secretory lung cancer subtype samples, respectively.

### Functional enrichment analysis

The gene ontology enrichment analysis were performed on all lncRNA-associated PPI modules from the overall generic lung cancer dataset, as well as the lung cancer subtypes, including classical, basal, and secretory. The Fisher exact test was used to calculate the statistical significance of each GO term, and then the *p*-values were corrected using the Benjamini-Hochberg procedure. The GO terms with corrected *p*-value < 0.05 were considered as enriched functions. The results of functional enrichment analysis can be found in the “Lncin” website which is freely accessible at http://lncin.ym.edu.tw/Case_Lung/Lung_result.php?id=cancer_lung#Predict.

For each lncRNA, the enriched functions (corrected *p*-value < 0.05) were graphically organized into a network, where each GO term is a node and edges represent gene overlap between GO terms^45^. The gene overlap was scored by the arithmetic average of Jaccard coefficient (JC) and Simpson coefficient (SC) defined as follows:


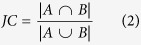



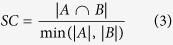


where *A* and *B* are two gene-sets. An edge which overlap score passes a threshold was presented in the networks. The networks were visualized by Cytoscape^46^.

### Functional similarities of a gene set

For a gene set *G* = (*g*_*1*_, *g*_*2*_, …, *g*_*n*_), the functional homogeneity of *G, fm,* was defined as:


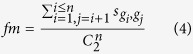


where *s* is the functional similarity between *g*_*i*_ and *g*_*j*_ based on GO annotation, and *C* is the combination function. The functional similarity was calculated using the R package GoSemSim^20^ with the “Rel” method to measure semantic similarity and the “BMA” method to combine the scores.

### Interaction density of a gene set

For a gene set *G* = (*g*_*1*_, *g*_*2*_, …, *g*_*n*_), the interaction density of *G, d,* was defined as:


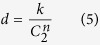


where *k* is the number of interaction existing among the gene set and *C* is the combination function.

### Clustering of lncRNA modules

The association between lncRNAs was assessed by calculating the overlap between lncRNA-associated PPI modules. The association index between lncRNA A and B was calculated by:


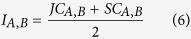


where *JC* and *OC* are the Jaccard coefficient and Simpson coefficient defined as:


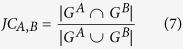



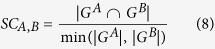


where *G*^*A*^ and *G*^*B*^ denote the associated mRNAs of lncRNA *A* and *B*, respectively. This association index was used as the similarity between lncRNAs, and the average-linkage hierarchical clustering was performed using GAP (Generalized Association Plots)^47^.

## Additional Information

**How to cite this article**: Wu, C.-H. *et al.* Identification of lncRNA functions in lung cancer based on associated protein-protein interaction modules. *Sci. Rep.* **6**, 35939; doi: 10.1038/srep35939 (2016).

**Publisher’s note:** Springer Nature remains neutral with regard to jurisdictional claims in published maps and institutional affiliations.

## Supplementary Material

Supplementary Information
