# Supplementary material for: Identification of lncRNA functions in lung cancer based on associated protein-protein interaction modules
Source: Sci Rep. 2016 Oct 27;6:35939. doi: 10.1038/srep35939 (PMC5081511; doi:10.1038/srep35939)
Supplement: Supplementary Information [file srep35939-s1.doc]

**Supplementary Information**

**Identification of lncRNA functions in lung cancer based on associated protein-protein interaction modules**

**Chih-Hsun Wu, Chia-Lang Hsu, Pei-Chun Lu,** **Wen-Chang Lin, Hsueh-Fen Juan, Hsuan-Cheng Huang**

This document contains Supplementary Figures S1-S10 and Tables S1-S2.


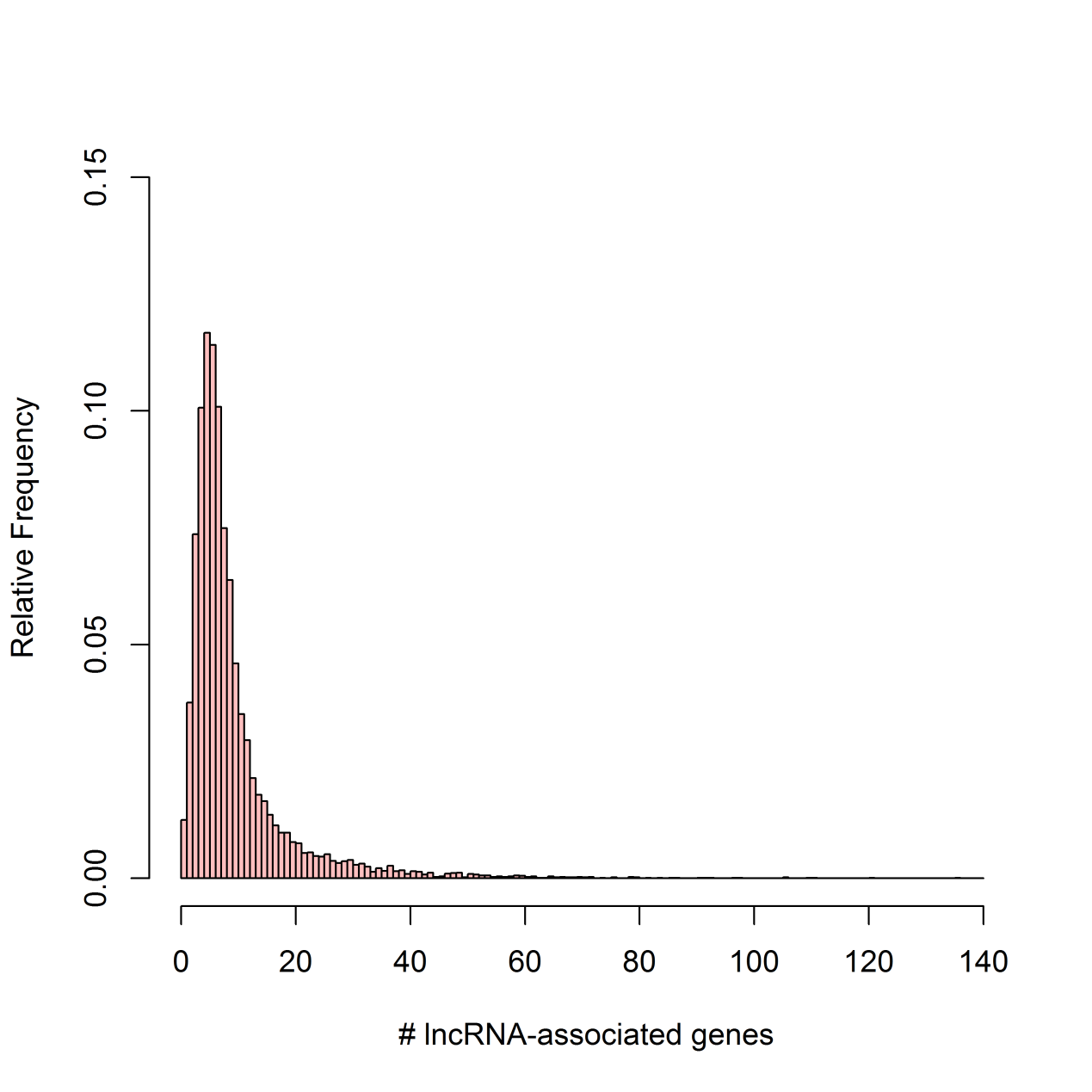
**#** lncRNA-co-expressed mRNAs

**Figure S1 - The distribution of co-expressed mRNAs of all lncRNAs**

For each lncRNA, the mRNAs with mutual rank <13.6 were considered as its coexpressed mRNAs. The figure shows the numbers of lncRNA-co-expressed mRNAs for all lncRNAs.

**
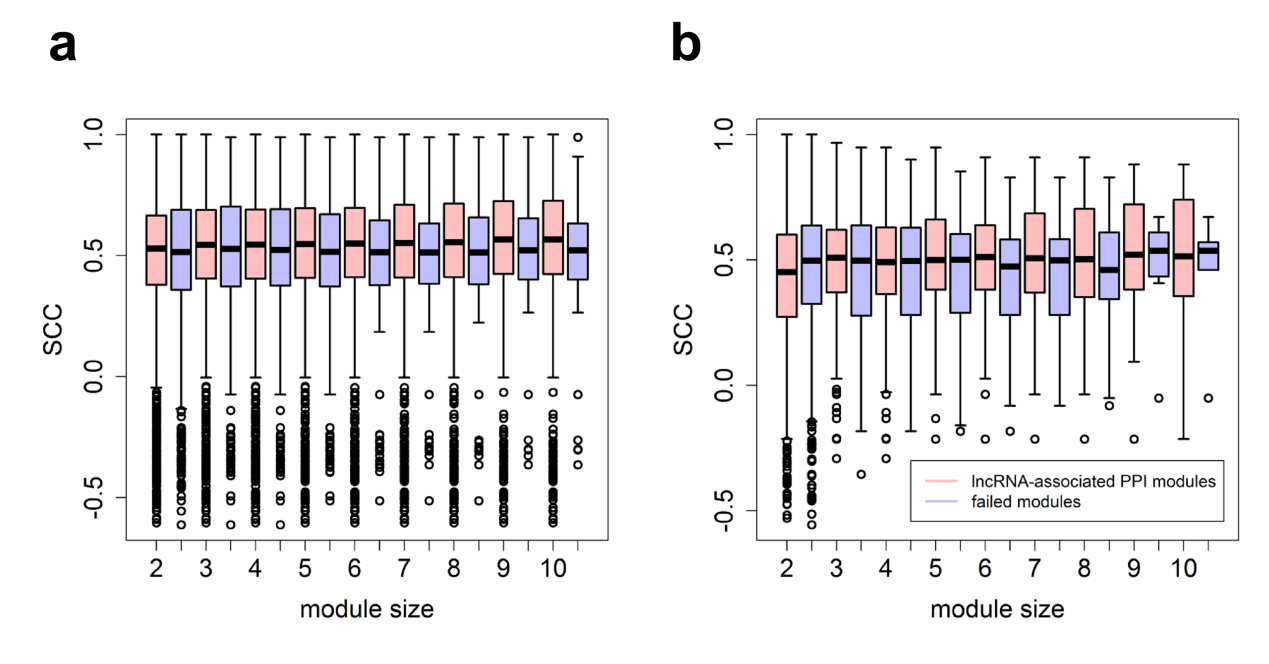
**

**Figure S2 - The distribution of expression correlation between lncRNA-associated PPI dense modules and failed modules**

(a) With various module sizes, we compared the distribution of expression correlation using SCC between mRNA-mRNA pairs of lncRNA-associated PPI modules (red) and mRNA-mRNA pairs of failed modules (a permutation test value of *p* > 0.05) (blue). (b) For each lncRNA, mean of SCC in mRNA-mRNA pairs of lncRNA-associated PPI modules and failed modules with various module sizes.


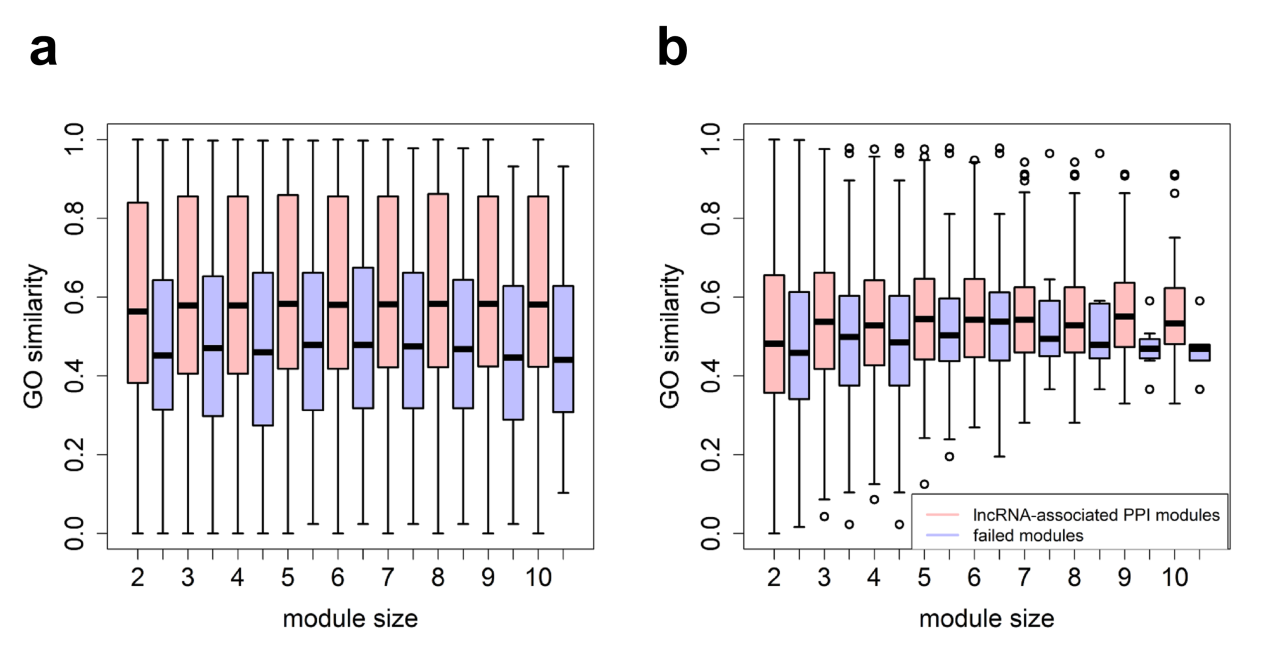


**Figure S3 - The distribution of functional consistency between lncRNA-associated PPI modules and failed modules**

(a) With various module sizes, we compared the distribution functional consistency using GO similarity between mRNA-mRNA pairs of lncRNA-associated PPI modules (red) and mRNA-mRNA pairs of failed modules (a permutation test value of *p* > 0.05) (blue). (b) For each lncRNA, mean of GO similarity in mRNA-mRNA pairs of lncRNA-associated PPI modules and failed modules with various module sizes.


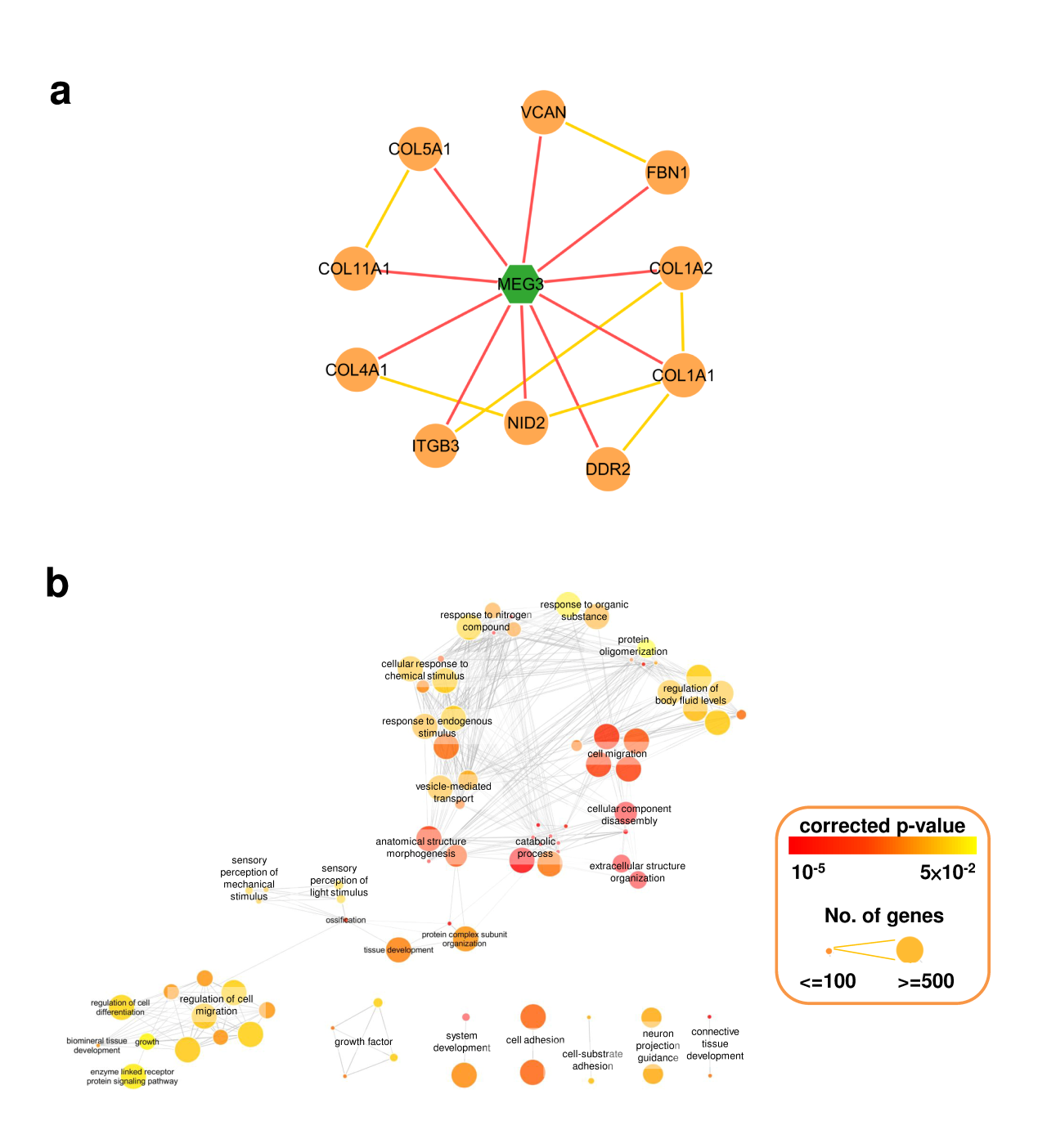


**Figure S4 – MEG3-associated PPI module and its enriched functions in classical LSCC**

(a) The MEG3-associated PPI module is visualized as a graph. Hexagon and oval node denote lncRNA and mRNA. Edge color represents different interaction type. (b) Enriched GO terms derived from the MEG3-associated PPI module are visualized as a network. Nodes represent enriched GO terms (corrected *p* < 0.05) and links between the nodes represent the overlap score calculated from the number of genes two GO terms share (threshold = 0.85). Node color encodes the statistical significance of enrichment analysis. The node size is proportional to the number of genes belonging to the corresponding GO term.

**
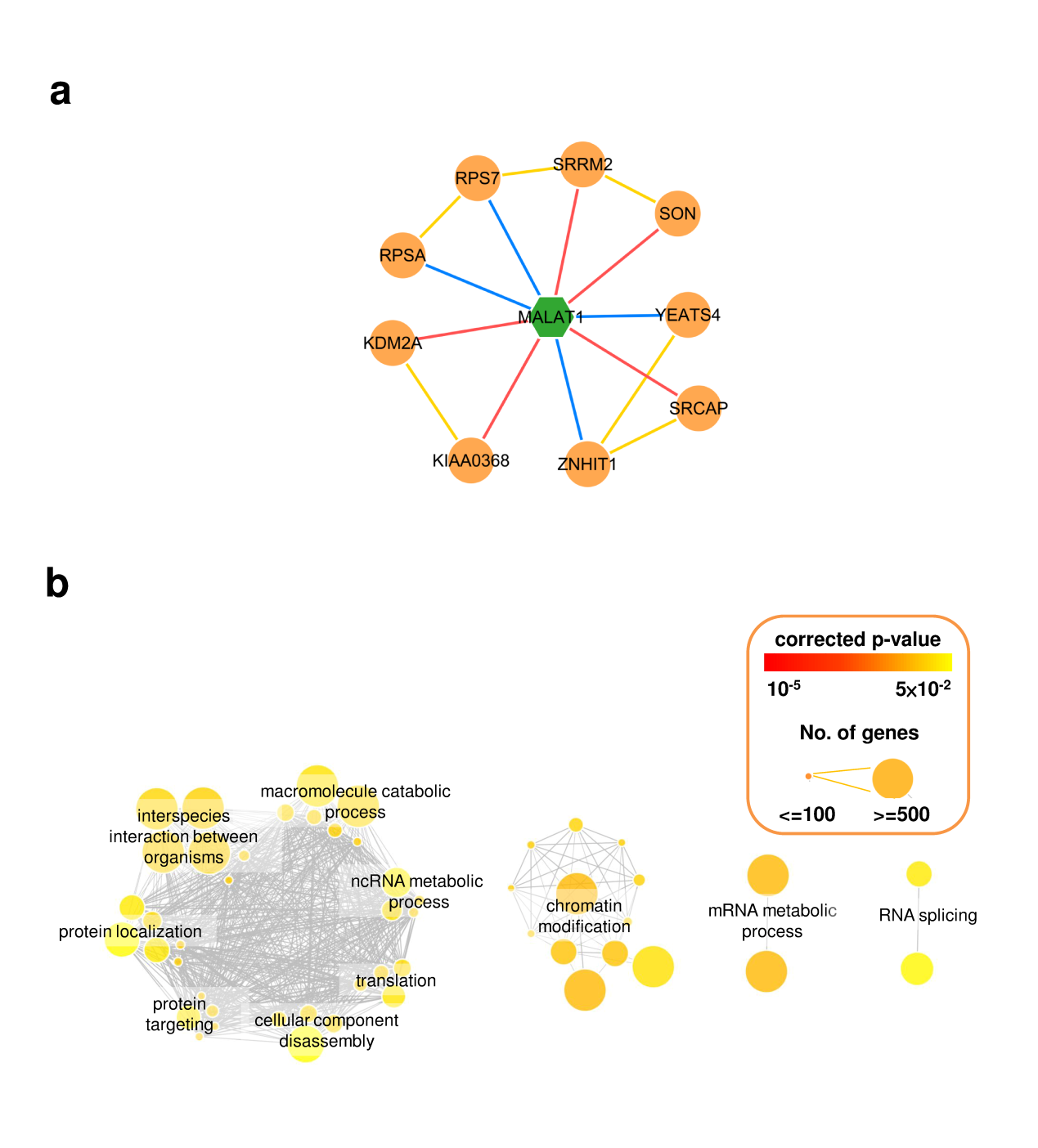
Figure S5 - MALAT1-associated PPI module and its enriched functions in classical LSCC**

(a) The MALAT1-associated PPI module is visualized as a graph. Hexagon and oval node denote lncRNA and mRNA. Edge color represents different interaction type. (b) Enriched GO terms derived from the MALAT1-associated PPI module are visualized as a network. Nodes represent enriched GO terms (corrected *p* < 0.05) and links between the nodes represent the overlap score calculated from the number of genes two GO terms share (threshold = 0.85). Node color encodes the statistical significance of enrichment analysis. The node size is proportional to the number of genes belonging to the corresponding GO term.

**
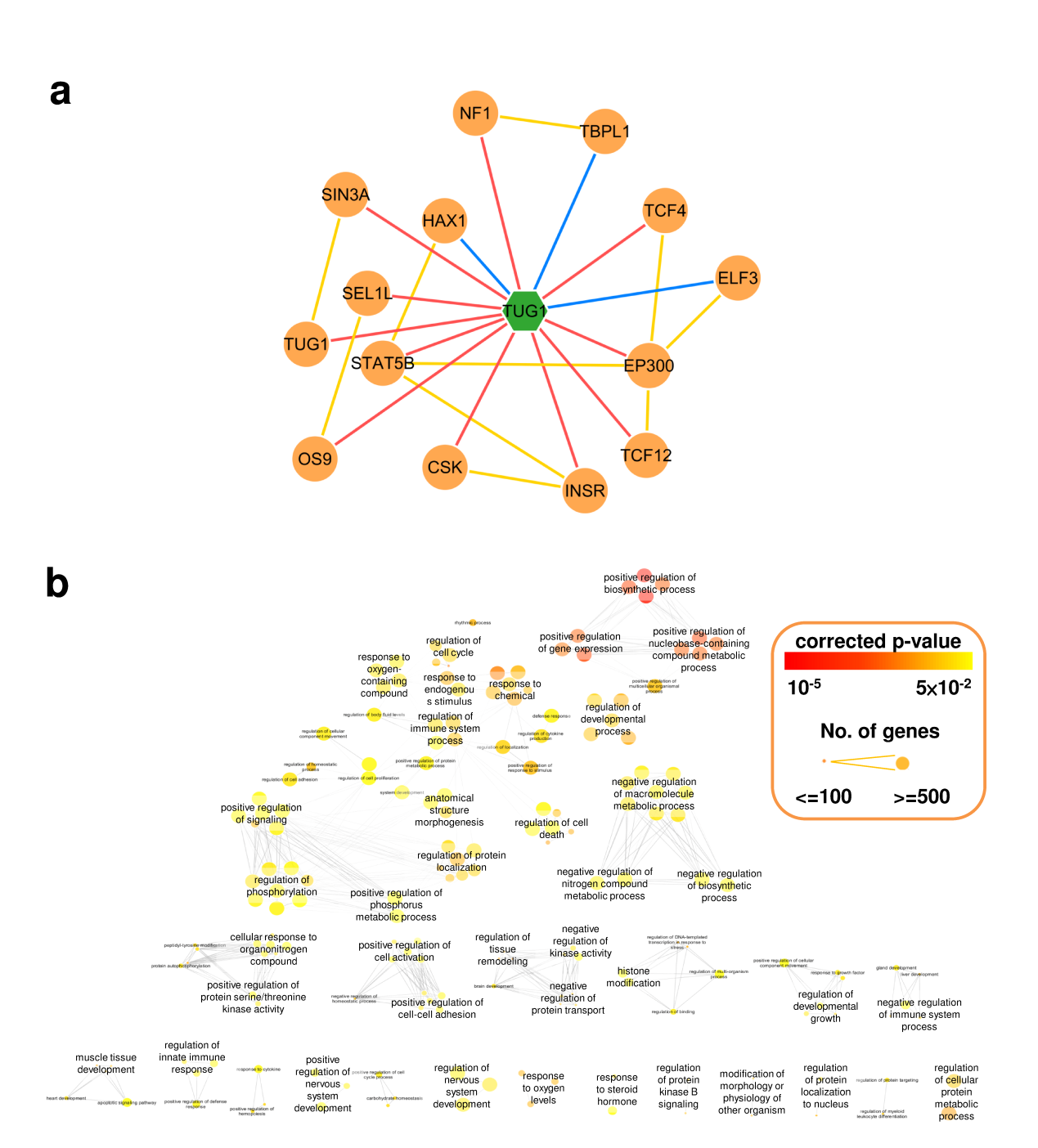
Figure S6 – TUG1-associated PPI module and its enriched functions in classical LSCC**

(a) The TUG1-associated PPI module is visualized as a graph. Hexagon and oval node denote lncRNA and mRNA. Edge color represents different interaction type. (b) Enriched GO terms derived from the TUG1-associated PPI module are visualized as a network. Nodes represent enriched GO terms (corrected p-value < 0.05) and links between the nodes represent the overlap score calculated from the number of genes two GO terms share (threshold = 0.85). Node color encodes the statistical significance of enrichment analysis. The node size is proportional to the number of genes belonging to the corresponding GO term. **
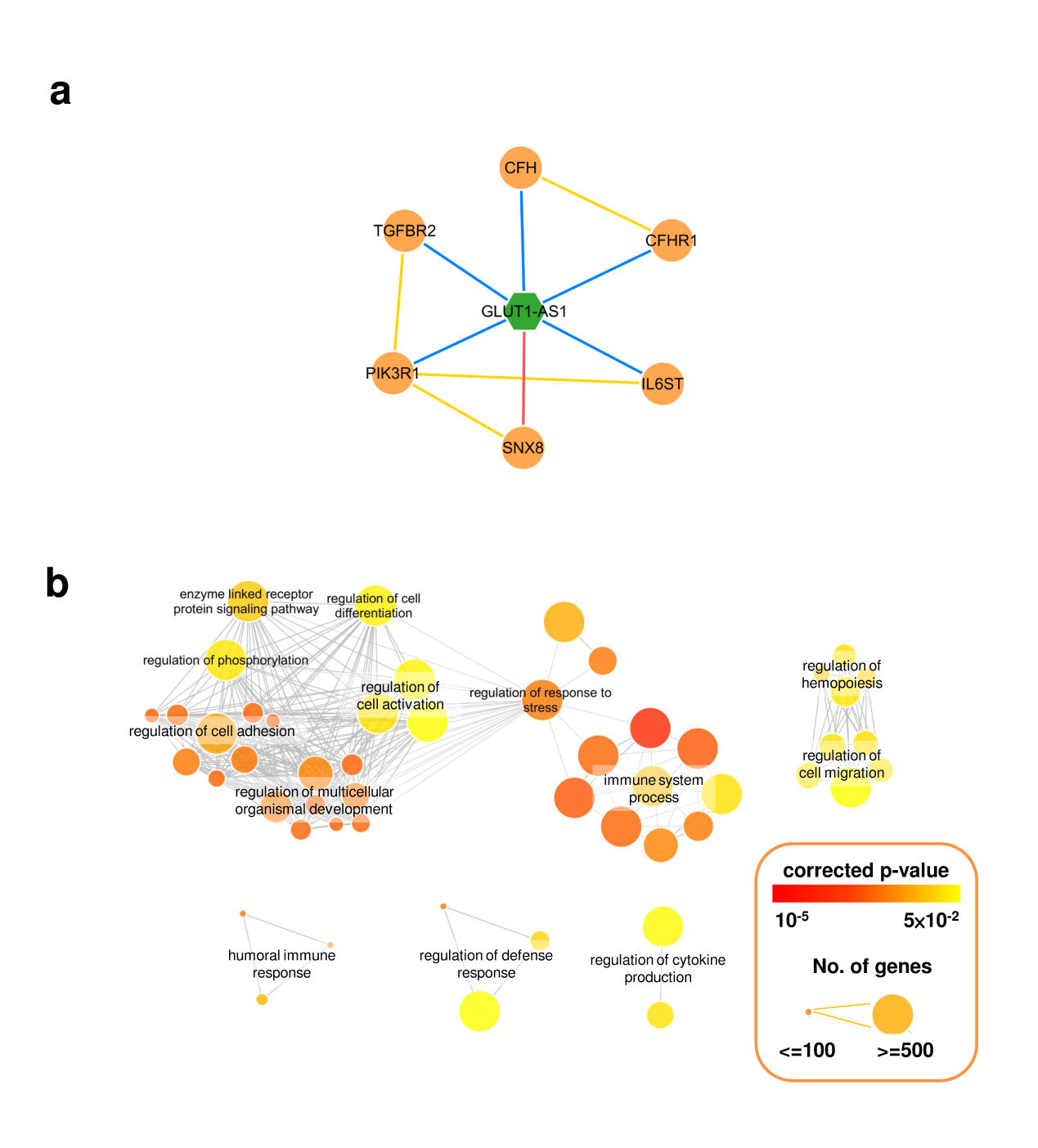
Figure S7 – GLUT1-AS1-associated PPI module and its enriched functions in secretory LSCC**

(a) The GLUT1-AS1-associated PPI module is visualized as a graph. Hexagon and oval node denote lncRNA and mRNA. Edge color represents different interaction type. (b) Enriched GO terms derived from the GLUT1-AS1-associated PPI module are visualized as a network. Nodes represent enriched GO terms (corrected *p* < 0.05) and links between the nodes represent the overlap score calculated from the number of genes two GO terms share (threshold = 0.85). Node color encodes the statistical significance of enrichment analysis. The node size is proportional to the number of genes belonging to the corresponding GO term.


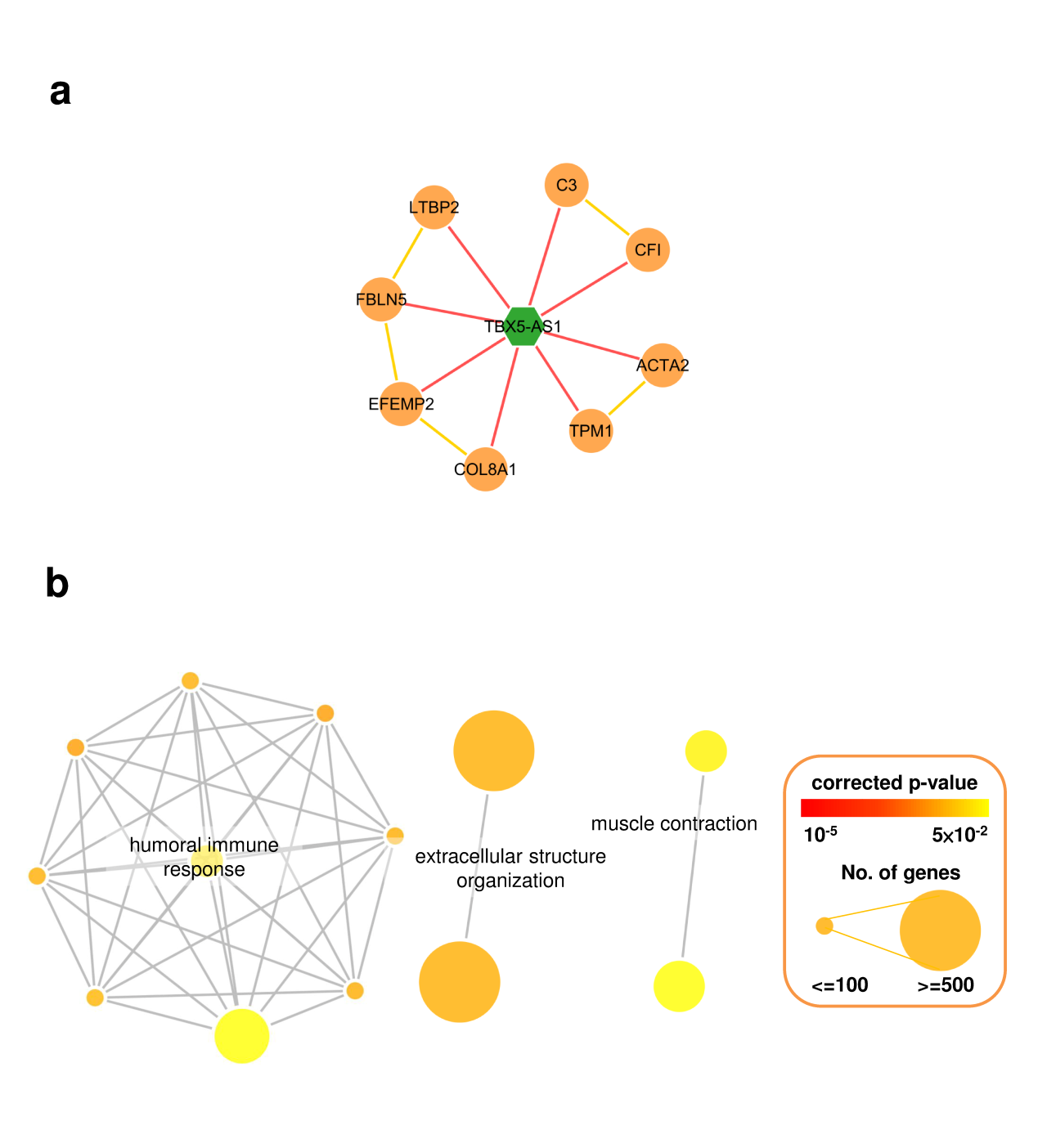
**Figure S8 – TBX5-AS1-associated PPI module and its enriched functions in classical LSCC**

(a) The TBX5-AS1-associated PPI module is visualized as a graph. Hexagon and oval node denote lncRNA and mRNA. Edge color represents different interaction type. (b) Enriched GO terms derived from the TBX5-AS1-associated PPI module are visualized as a network. Nodes represent enriched GO terms (corrected *p* < 0.05) and links between the nodes represent the overlap score calculated from the number of genes two GO terms share (threshold = 0.85). Node color encodes the statistical significance of enrichment analysis. The node size is proportional to the number of genes belonging to the corresponding GO term.


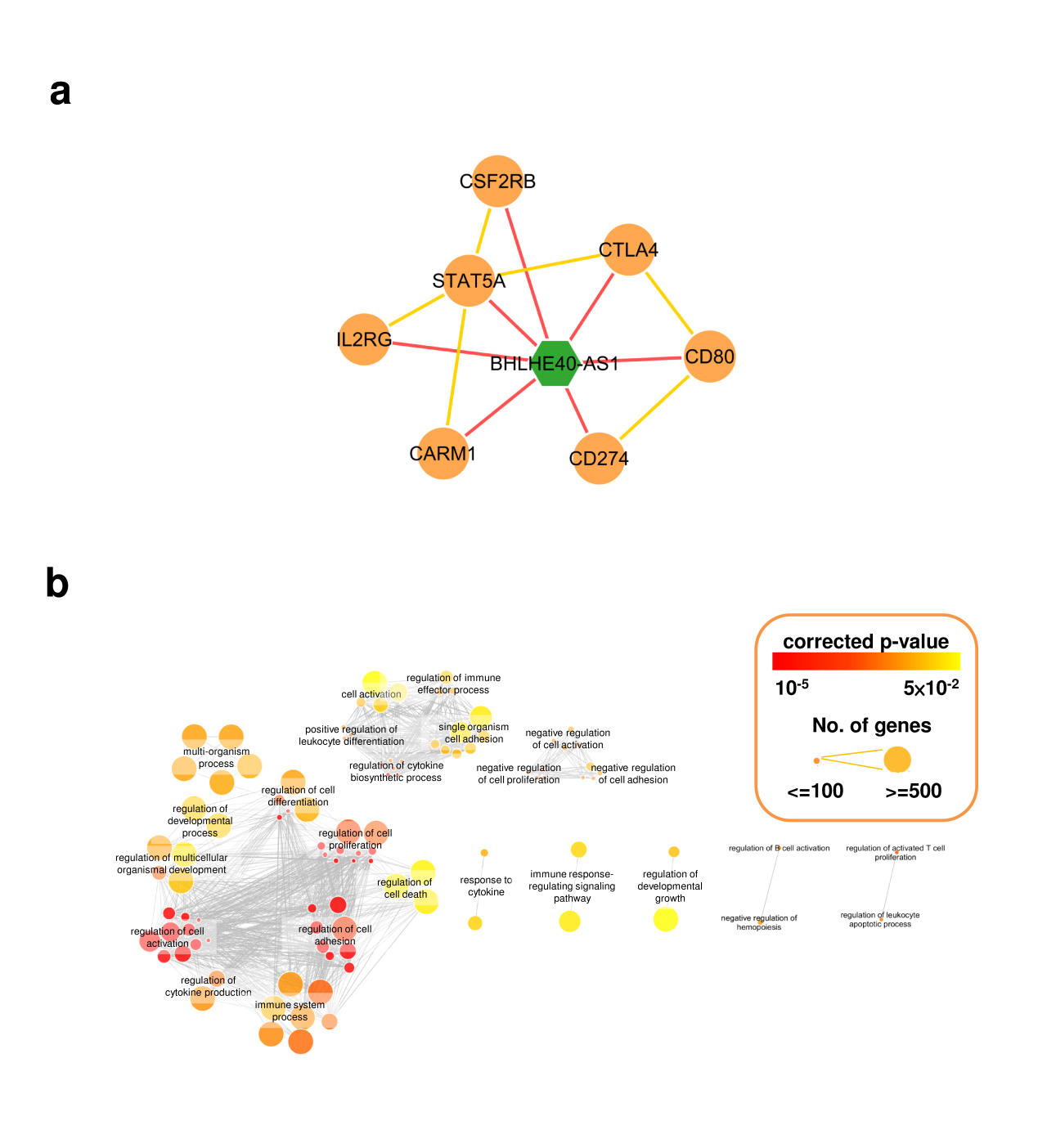
**Figure S9 – BHLHE40-AS1-associated PPI module and its enriched functions in secretory LSCC**

(a) The BHLHE40-AS1-associated PPI module is visualized as a graph. Hexagon and oval node denote lncRNA and mRNA. Edge color represents different interaction type. (b) Enriched GO terms derived from the BHLHE40-AS1-associated PPI module are visualized as a network. Nodes represent enriched GO terms (corrected *p* < 0.05) and links between the nodes represent the overlap score calculated from the number of genes two GO terms share (threshold = 0.85). Node color encodes the statistical significance of enrichment analysis. The node size is proportional to the number of genes belonging to the corresponding GO term.

**
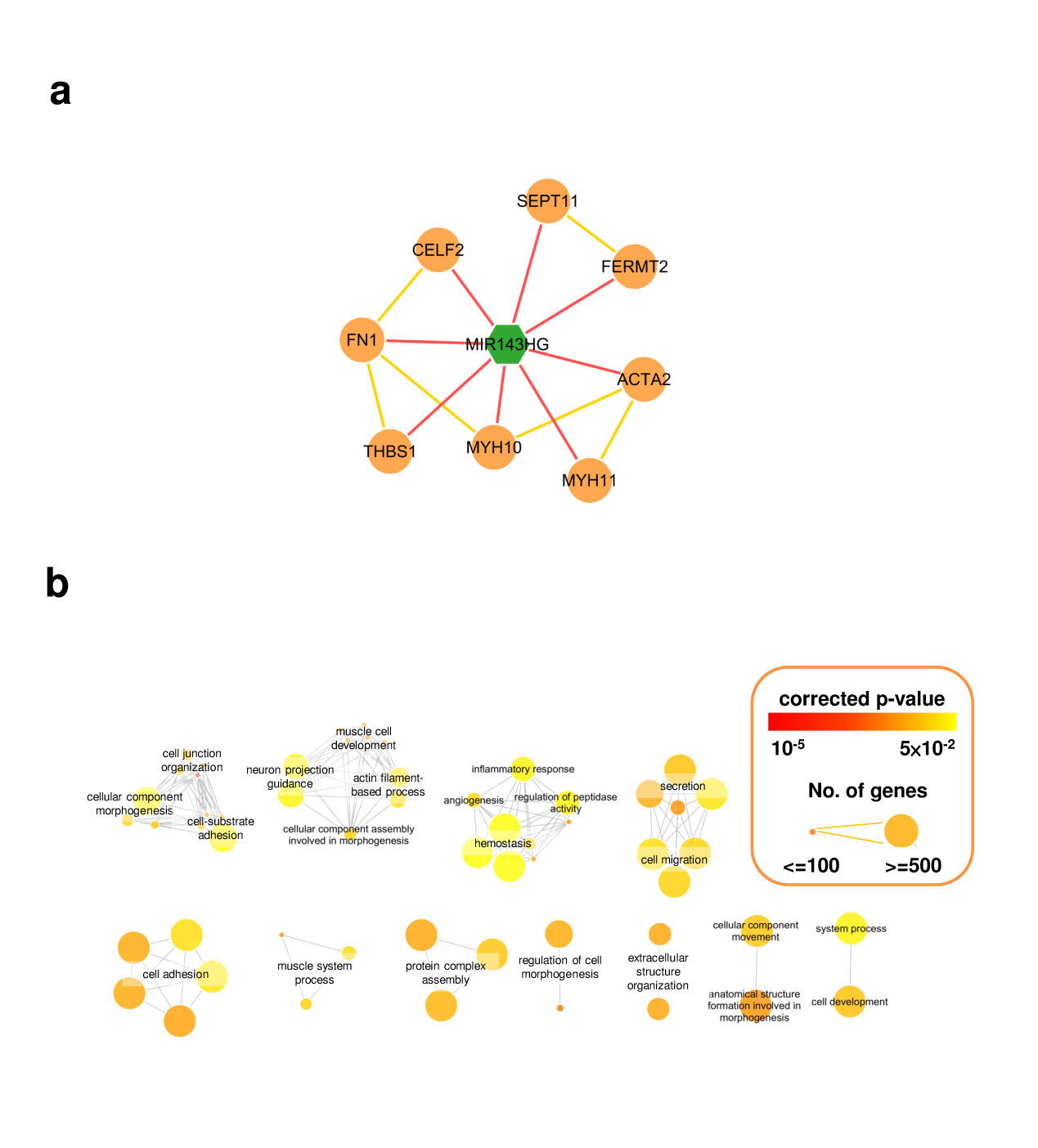
Figure S10 – MIR143HG-associated PPI module and its enriched functions in secretory LSCC**

(a) The MIR143HG-associated PPI module is visualized as a graph. Hexagon and oval node denote lncRNA and mRNA. Edge color represents different interaction type. (b) Enriched GO terms derived from the MIR143HG-associated PPI module are visualized as a network. Nodes represent enriched GO terms (corrected *p* < 0.05) and links between the nodes represent the overlap score calculated from the number of genes two GO terms share (threshold = 0.85). Node color encodes the statistical significance of enrichment analysis. The node size is proportional to the number of genes belonging to the corresponding GO term.

**Table S1 - The functions of 18 well-studied lncRNAs from literature and our method**

We found 18 well-studied lncRNAs which were reported in literature and were also identified by our analysis in generic, classical, basal and secretory LUSC.

| **Subtype** | **lncRNA ID** | **lncRNA name** | **Functions from literature** | **Functions inferred by our method** | **Consistent** |
| --- | --- | --- | --- | --- | --- |
| generic | ENSG00000172965 | MIR4435-2HG | Oncogene, cell growth inhibition, cell cycle (lung cancer), stem cell differentiation | regulation of cell cycle, somatic stem cell maintenance, chromatin modification, RNA splicing | V |
| classical |  |  |  | stem cell maintenance, negative regulation of cell differentiation, RNA splicing |  |
| basal |  |  |  | cell cycle checkpoint, RNA splicing |  |
| generic | ENSG00000175061 | LRRC75A-AS1(C17orf76-AS1) | tumor suppressor-activated pathway, intracellular signal transduction, epidermal growth factor and extracellular signal-regulated kinase (EGF/ERK) signaling cascade | positive regulation of apoptotic signaling pathway, gene expression, ncRNA metabolic process, ribonucleoprotein complex subunit organization | V |
| generic | ENSG00000186594 | MIR22HG | chemical stress, cellular stress responses | cellular response to stress, cell cycle | V |
| generic | ENSG00000197989 | SNHG12 | cell proliferation, cell apoptosis, cell cycle (endometrial carcinoma) | cell cycle process, RNA splicing | V |
| generic | ENSG00000214548 | MEG3 | putative tumor suppressor, activation of p53, inhibition of cell proliferation, growth, development | regulation of cell proliferation | V |
| classical |  |  |  | extracellular structure organization, cell migration, cell adhesion, growth |  |
| generic | ENSG00000215417 | MIR17HG | cell cycle, cell survival, proliferation, differentiation, angiogenesis | cell cycle phase transition, RNA splicing | V |
| generic | ENSG00000224032 | Transcript Induced By Growth Arrest 1 (EPB41L4A-AS1) | growth arrest, cell proliferation, inorganic ion transport, metabolism,vascular calcification | organic substance transport, mRNA metabolic process, ncRNA metabolic process | V |
| generic | ENSG00000230838 | AC093850.2 | tumor stage, proliferation, cell cycle, epigenetic suppression (lung cancer) | extracellular structure organization, biological adhesion, cell migration | V |
| basal |  |  |  | extracellular structure organization, biological adhesion, cell migration |  |
| generic | ENSG00000234883 | MIR155HG | B-cell receptor signaling, B-cell receptor activation (chronic lymphocytic leukemia,Hodgkin lymphoma), T cell activation, innate immunity, cell cycle (lung cancer) | B cell receptor signaling pathway, B cell activation, T cell activation, innate immune response | V |
| classical | [ENSG00000243197](http://lncin.ym.edu.tw/Case_Lung/Lung_enrichment.php?id=cancer_lung&lncRNA=classical-ENSG00000243197(LSAMP-AS1)) | Tumor Suppressor Candidate 7 (LSAMP-AS1) | p53-regulated tumor suppressor (osteosarcoma, colorectal cancer), cell growth inhibition(colorectal cancer), prognosis (colorectal cancer, osteosarcoma, gastric cancer), chemoradiotherapy resistance and poor prognosis (esophageal squamous cell carcinoma), proliferation of normal osteoblasts by cell-cycle transcripts (Cyclin D1, VEGF, and VEGFR1 up-regulation, and cyclin A2 and cyclin B1 suppression) and regulation of apoptotic genes | positive regulation of cell cycle process, regulation of I-kappaB kinase/NF-kappaB signaling | V |
| basal | [ENSG00000244383](http://lncin.ym.edu.tw/Case_Lung/Lung_enrichment.php?id=cancer_lung&lncRNA=basal-ENSG00000244383) | FAM3D-AS1 | survival, prognostic factors, stem cell and EMT gene expression, migration, proliferation (head and neck squamous cell carcinoma) | RNA splicing, peptidyl-amino acid modification | X |
| generic | ENSG00000245532 | NEAT1 | paraspeckle formation, cancer progression, chromatin modification, mRNA splicing | covalent chromatin modification, mRNA splicing | V |
| generic | ENSG00000245910 | SNHG6 | ribosomes-related, resistant to nonsense-mediated mRNA decay | ribonucleoprotein complex subunit organization, nuclear-transcribed mRNA catabolic process, nonsense-mediated decay | V |
| secretory | [ENSG00000249669](http://lncin.ym.edu.tw/Case_Lung/Lung_enrichment.php?id=cancer_lung&lncRNA=secretory-ENSG00000249669(MIR143-HG)) | MIR143-HG | regulator of cardiac cell differentiation and homeostasis | cardiac cell development, cell morphogenesis involved in differentiation, extracellular matrix organization, cell adhesion | V |
| generic | ENSG00000249859 | PVT1 | proliferation and stem cell-like property, regulation by tumor suppressor p53, regulation of proto-oncogene MYC, candidate oncogene | regulation of stem cell proliferation, chromatin modification, cell cycle | V |
| classical | [ENSG00000250266](http://lncin.ym.edu.tw/Case_Lung/Lung_enrichment.php?id=cancer_lung&lncRNA=classical-ENSG00000250266) | LINC01612 | metastasis, TNM (tumour, node, and metastasis) stage, survival (gastric cancer) | No reriched functions | X |
| classical | [ENSG00000251562](http://lncin.ym.edu.tw/Case_Lung/Lung_enrichment.php?id=cancer_lung&lncRNA=classical-ENSG00000251562(MALAT1)) | MALAT1 | oncogene, tumorigenesis, regulation of gene expression, chromatin modification | chromatin modification, RNA splicing | V |
| generic | ENSG00000253352 | TUG1 | cell proliferation (ostearcoma, non-small cell lung cancer, esophageal squamous cell carcinoma, HCC), migration (esophageal squamous cell carcinoma), cell cycle, apoptosis (HCC) | cell cycle process | V |
| classical |  |  |  | regulation of cell proliferation, positive regulation of growth, regulation of the cell cycle, regulation of cell adhesion |  |

**Table S2 - The similarity of lncRNA-associated genes and functional similarity of common lncRNAs in different subtypes**

To compare the components of common lncRNA-associated PPI modules, the similarity of lncRNA-associated genes was calculated. To investigate the functions of common lncRNAs in different subtypes, we selected GO terms (p<0.05, n<1000) and used R package GO semsim to compare the GO terms of common lncRNAs.

| **Common lncRNAs between generic and classical subtype** | | | | | | | |  | |  | |  | |  | |  |
| --- | --- | --- | --- | --- | --- | --- | --- | --- | --- | --- | --- | --- | --- | --- | --- | --- |
| lncRNA | | No. components in generic samples | | No. components in classical samples | | No. common components | | Jaccard index | | Overlap index | | (Jaccard + Overlap)/2 | | Function similarity score | |  |
| ENSG00000214548 (MEG3) | | 7 | | 10 | | 3 | | 0.21 | | 0.43 | | 0.32 | | 0.84 | |  |
| ENSG00000223675 | | 6 | | 7 | | 5 | | 0.63 | | 0.83 | | 0.73 | | 0.85 | |  |
| ENSG00000233093 | | 10 | | 8 | | 0 | | 0.00 | | 0.00 | | 0.00 | | 0.84 | |  |
| ENSG00000237265 | | 12 | | 6 | | 2 | | 0.13 | | 0.33 | | 0.23 | | 0.93 | |  |
| ENSG00000245970 | | 11 | | 7 | | 4 | | 0.29 | | 0.57 | | 0.43 | | 0.90 | |  |
| ENSG00000253352 (TUG1) | | 29 | | 14 | | 1 | | 0.02 | | 0.07 | | 0.05 | | 0.60 | |  |
| ENSG00000255399 (TBX5-AS1) | | 20 | | 8 | | 2 | | 0.08 | | 0.25 | | 0.16 | | 0.35 | |  |
| ENSG00000259939 | | 8 | | 18 | | 1 | | 0.04 | | 0.13 | | 0.08 | | 0.58 | |  |
| ENSG00000260032 | | 20 | | 16 | | 3 | | 0.09 | | 0.19 | | 0.14 | | 0.63 | |  |
| XLOC_013174 | | 20 | | 19 | | 1 | | 0.03 | | 0.05 | | 0.04 | | 0.65 | |  |
| **Common lncRNAs between generic and basal subtype** | | | | | | | |  | |  | |  | |  | |  |
| lncRNA | | No. components in generic samples | | No. components in basal  samples | | No.  common components | | Jaccard index | | Overlap index | | (Jaccard + Overlap)/2 | | Function similarity score | |  |
| ENSG00000230838 | | 14 | | 23 | | 9 | | 0.32 | | 0.64 | | 0.48 | | 0.90 | |  |
| ENSG00000231486 | | 10 | | 11 | | 8 | | 0.62 | | 0.80 | | 0.71 | | 0.81 | |  |
| ENSG00000249548 | | 15 | | 10 | | 7 | | 0.39 | | 0.70 | | 0.54 | | 0.92 | |  |
| ENSG00000249835 (VCAN-AS1) | | 8 | | 12 | | 5 | | 0.33 | | 0.63 | | 0.48 | | 0.80 | |  |
| ENSG00000256262 | | 15 | | 9 | | 2 | | 0.09 | | 0.22 | | 0.16 | | 0.65 | |  |
| ENSG00000258135 | | 21 | | 9 | | 0 | | 0.00 | | 0.00 | | 0.00 | | 0.64 | |  |
| ENSG00000259234 | | 10 | | 8 | | 6 | | 0.50 | | 0.75 | | 0.63 | | 0.84 | |  |
| ENSG00000260565 | | 9 | | 11 | | 4 | | 0.25 | | 0.44 | | 0.35 | | 0.54 | |  |
| XLOC_003405 | | 9 | | 7 | | 1 | | 0.07 | | 0.14 | | 0.10 | | 0.72 | |  |
| XLOC_005690 | | 7 | | 6 | | 0 | | 0.00 | | 0.00 | | 0.00 | | 0.56 | |  |
| XLOC_009913 | | 19 | | 6 | | 1 | | 0.04 | | 0.17 | | 0.10 | | 0.76 | |  |
| **Common lncRNAs between generic and secretory subtype** | | | | | | | |  | |  | |  | |  | |  |
| lncRNA | | No. components in generic samples | | No. components in secretory samples | | No. common components | | Jaccard index | | Overlap index | | (Jaccard + Overlap)/2 | | Function similarity score | |  |
| ENSG00000228741 | | 15 | | 20 | | 8 | | 0.30 | | 0.53 | | 0.41 | | 0.89 | |  |
| ENSG00000232533 | | 11 | | 6 | | 1 | | 0.06 | | 0.17 | | 0.11 | | 0.45 | |  |
| ENSG00000234184 | | 11 | | 6 | | 4 | | 0.31 | | 0.67 | | 0.49 | | 0.45 | |  |
| ENSG00000234961 | | 22 | | 7 | | 4 | | 0.16 | | 0.57 | | 0.37 | | 0.68 | |  |
| ENSG00000236481 | | 7 | | 8 | | 3 | | 0.25 | | 0.43 | | 0.34 | | 0.78 | |  |
| ENSG00000249178 | | 11 | | 13 | | 5 | | 0.26 | | 0.45 | | 0.36 | | 0.75 | |  |
| **Common lncRNAs between classical and secretory subtype** | | | | | | | |  | |  | |  | |  | |  |
| lncRNA | | No. components in classical samples | | No. components in secretory samples | | No. common components | | Jaccard index | | Overlap index | | (Jaccard + Overlap)/2 | | Function similarity score | |  |
| ENSG00000261039 | | 11 | | 10 | | 0 | | 0.00 | | 0.00 | | 0.00 | | 0.59 | |  |
| **Common lncRNAs among generic, classical and secretory subtype** | | | | | | |  | |  | |  | |  | |  | |
| lncRNA | No. components in generic samples | | No. components in classical samples | | No. components in secretory samples | | No.  common components | | Jaccard index | | Overlap index | | (Jaccard + Overlap)/2 | | Function similarity score | |
| ENSG00000235979 | 34 | | 26 | | 23 | | 9 | | 0.12 | | 0.39 | | 0.26 | | 0.87 | |
| ENSG00000253830 | 7 | | 6 | | 6 | | 0 | | 0.00 | | 0.00 | | 0.00 | | 0.36 | |
| **Common lncRNAs among generic, classical and basal subtype** | | | | | | |  | |  | |  | |  | |  | |
| lncRNA | No. components in generic samples | | No. components in classical samples | | No. components in basal  samples | | No.  common components | | Jaccard index | | Overlap index | | (Jaccard + Overlap)/2 | | Function similarity score | |
| ENSG00000172965 (MIR4435-2HG) | 41 | | 7 | | 13 | | 1 | | 0.02 | | 0.14 | | 0.08 | | 0.50 | |
| ENSG00000235576 | 19 | | 10 | | 7 | | 4 | | 0.13 | | 0.57 | | 0.35 | | 0.84 | |
| ENSG00000247774 | 24 | | 16 | | 10 | | 2 | | 0.04 | | 0.20 | | 0.12 | | 0.63 | |
| ENSG00000255471 | 16 | | 6 | | 6 | | 0 | | 0.00 | | 0.00 | | 0.00 | | 0.50 | |
| ENSG00000256039 | 16 | | 14 | | 7 | | 1 | | 0.03 | | 0.14 | | 0.09 | | 0.85 | |
